# Transplantation of maternal intestinal flora to the newborn after elective cesarean section (SECFLOR): study protocol for a double blinded randomized controlled trial

**DOI:** 10.1186/s12887-022-03609-3

**Published:** 2022-09-29

**Authors:** Noora Carpén, Petter Brodin, Willem M. de Vos, Anne Salonen, Kaija-Leena Kolho, Sture Andersson, Otto Helve

**Affiliations:** 1 Pediatric Research Center, New Children’s Hospital, University of Helsinki and Helsinki University Hospital, Biomedicum, Stenbäckinkatu 9, Haartmaninkatu 8, P.O. Box 63, 00014 Helsinki, Finland; 2 SciLifeLab, Department of Women’s and Children’s Health, Karolinska Institutet, K6 Kvinnors och barns hälsa, K6 Klinisk pediatrik, 171 77 Stockholm, Sweden; 3 Department of Immunology and Inflammation, Imperial College London, London, UK; 4 Department of Bacteriology and Immunology, Faculty of Medicine, Human Microbiome Research Program, University of Helsinki, PL 63 Haartmaninkatu 8, 00014 Helsinki, Finland; 5 Laboratory of Microbiology, Wageningen University, Wageningen, The Netherlands

**Keywords:** Microbiome, Oral fecal transplant, Newborn, Cesarean section, Development of immune system

## Abstract

**Background:**

A complication of elective cesarean section (CS) delivery is its interference with the normal intestinal colonization of the infant, affecting the immune and metabolic signaling in early life— a process that has been associated with long-term morbidity, such as allergy and diabetes. We evaluate, in CS-delivered infants, whether the normal intestinal microbiome and its early life development can be restored by immediate postnatal transfer of maternal fecal microbiota (FMT) to the newborn, and how this procedure influences the maturation of the immune system.

**Methods:**

Sixty healthy mothers with planned elective CS are recruited and screened thoroughly for infections. A maternal fecal sample is taken prior to delivery and processed according to a transplantation protocol. After double blinded randomization, half of the newborns will receive a diluted aliquot of their own mother’s stool orally administered in breast milk during the first feeding while the other half will be similarly treated with a placebo. The infants are clinically followed, and fecal samples are gathered weekly until the age of 4 weeks, then at the ages of 8 weeks, 3, 6, 12 and 24 months. The parents fill in questionnaires until the age of 24 months. Blood samples are taken at the age of 2–3 days and 3, 6, 12 and 24 months to assess development of major immune cell populations and plasma proteins throughout the first years of life.

**Discussion:**

This is the first study to assess long-time effects on the intestinal microbiome and the development of immune system of a maternal fecal transplant given to term infants born by CS.

**Trial registration:**

ClinicalTrials.gov NCT04173208, registration date 21.11.2019.

**Supplementary Information:**

The online version contains supplementary material available at 10.1186/s12887-022-03609-3.

## Background

Microbes colonizing newborn infants at birth and during the first weeks of life are considered important, and perturbation to this process of colonization is associated with disruption of the stereotypic immune system development early in life [1, 2]. Early disturbances in gut microbiome have been associated with a multitude of inflammatory diseases, such as the development of autoimmune diseases [3], allergic diseases and atopy [4, 5], asthma, type I diabetes [6], and inflammatory bowel diseases [7, 8]. A recently published population cohort study also showed that compared with vaginally-delivered (VD) infants, the risk of infection-related hospitalization was greater among those born by cesarean section (CS) [9]. The difference of the early colonizing microbiome was thought to be one of the associating factors [9, 10].

The first stools of the newborns, the meconium, contain very low amounts of bacterial DNA and their composition differs greatly from that of later stool samples. The stool microbial communities develop along similar and time-dependent trajectories, in the absence of treatment with antibiotics [11, 12]. During the postnatal period the gut microbiome of VD and by CS-delivered infants differs markedly from each other [12–16] and this difference persists throughout the first years of life [17]. The disturbed transmission of the maternal gastrointestinal bacteria (particularly pioneering *Bacteroides* and *Bifidobacterium* species) through delivery by CS and maternal intrapartum antibiotic prophylaxis predispose newborn infants to colonization by potentially clinically important opportunistic pathogens that circulate in the hospital environment [16]. Infants born by CS have a less diverse intestinal microbiome and lower Th1 response than those born by vaginal delivery [18].

Various studies show that the intestinal microbiome influences vaccine responses, especially early in life [19–21]. Pneumococcal vaccine response can be studied for example by measuring serotype specific IgG antibodies. Finnish children are vaccinated with the pneumococcal conjugate vaccine at the age of 3, 5 and 12 months.

Due to the anticipated impact of disturbed microbial development on the developing immune system associated with delivery by CS, many researchers have tried to alleviate the effect of CS delivery on child microbiota. Treating the mother and breastfed child with a multispecies probiotic supplement may correct undesired changes in microbiota composition and function caused by cesarean birth [22]. Breastfeeding has a significant role in shaping the microbiota for example because of its oligosaccharides. Fucosylated oligosaccharides in mother's milk may also alleviate the effects of cesarean birth on infant gut microbiota [23]. A recent study reported that the microbial composition of breast milk was associated with the birth mode and exposure to intrapartum antibiotics [24].

Maternal probiotic supplementation has been shown to decrease the development of atopic dermatitis in the offspring [25]. In a 5-year and later on 13-year follow-up, probiotic intervention protected CS-delivered children from allergic disease and eczema [26, 27]. However, the topic is controversial and there is lack of evidence that probiotics prevent other chronic immune-mediated diseases.

Vaginal microbial transfer has been suggested to partially restore the intestinal microbiome of CS-delivered infants [28]. However, the vaginal microbiome is limited to mainly *Lactobacillus spp*. and does not contain the microbes that are abundant in the gut microbiota of the infant or the mother [14]. In addition, vaginal lactobacilli do not colonize the gastrointestinal tract of neonates but only pass it transiently, and their relevance for long-term health remains unclear [14, 29]. Hence, we recently reported that a fecal transfer from the infant’s own mother could restore rapidly and significantly the microbiome of CS-delivered infants towards that of those born vaginally [30]. Among the 17 mothers recruited, seven were selected after careful screening. Their infants received a diluted fecal sample from their own mothers, taken prior to delivery. Following a 3-month follow-up, all seven infants presented an uneventful clinical course without any adverse effects. The temporal development of the fecal microbiota composition of FMT-treated CS-delivered infants no longer resembled that of untreated CS-delivered infants but showed significant similarity to that of VD infants.

The implication of this study is that maternal fecal microbiota transplantation (FMT) mimics the natural transfer of microbiota from mother to VD infants.

Here, we present a protocol for randomized, double-blinded placebo-controlled clinical trial of orally delivered FMT from mothers to their CS-delivered infants, with a two-year follow-up period. The aim of the trial is to study the differences in microbiota between infants born by CS that received maternal fecal microbiota or a placebo transplantation, and to gain insight into the mechanisms involved in immune development and its association with early microbiota composition.

## Significance

### Study purpose

To the best of our knowledge, this is the first randomized study to assess whether maternal fecal transplant can be used as a method to close the gap in the heterogeneity of the intestinal microbiota of CS-delivered infants compared with the VD infants. In addition, it gives a unique possibility to study the interaction of gastrointestinal microbiome with the development of immunity during infancy.

## Study design

**Fig. 1:**
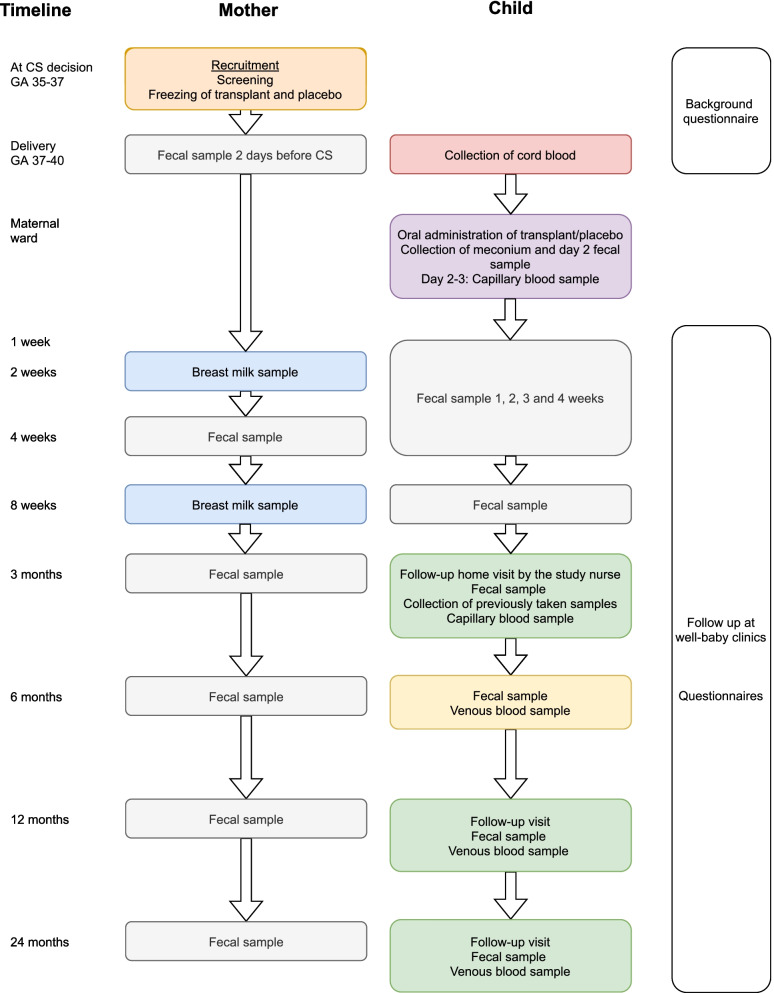
Flowchart of SECFLOR Main Study

One hundred healthy pregnant women scheduled for elective CS at term, are recruited at 35–37 weeks of gestation during a visit for the assessment of mode of delivery (Fig. 1) at Women’s Hospital, Helsinki and Jorvi Hospital, Espoo, Finland. Maternal exclusion criteria are age below 18 years, gestational diabetes that requires medication, use of regular medication, known or suspected fetal congenital abnormality, travelling abroad within the last three months and antibiotic treatment within 3 months of delivery (excluding the prophylactic cefuroxime (or other in case of allergy) given prior to the elective CS). Non-elective CS is an exclusion criterion.

The midwifes and doctors inform potential mothers of the study at the visit and those who are interested to participate are contacted by the study nurse.

After informed consent, the pregnant woman is tested for potential contagious diseases as explained below (Table 1). Resulting from the screening process, a 40% exclusion rate is expected because of *group B streptococci* (GBS) carrier state (24% of pregnant women in our region) [31] and other positive findings in the screening samples.

**Table 1:** Screening samples from the mother

**Blood tests**
Complete Blood Count, Erythrocyte sedimentation rate, C-reactive protein, Alanine transaminase, Alkaline phosphatase, Gamma-glutamyl transferase, Albumin, Creatinine, HIV, Hepatitis B, C and E, Treponema pallidum, Human T-cell leukemia virus
**Vaginal and perineal screening**
Group B *Streptococcus*, Methicillin-resistant *Staphylococcus aureus* (MRSA), Extended Spectrum Beta Lactamase (ESBL)
**Fecal screening**
* Salmonella, Shigella, Campylobacter, Yersinia enterocolitica/pseudotuberculosis,* Enterohemorragic *E. coli* (EHEC), Enteroaggregative *E. coli* (EAEC), Enteroinvasive *E. coli* (EIEC), Enterotoxigenic *E. coli* (ETEC), Enteropatogenic *E. coli* (EPEC), *Clostridium difficile*, Parasites, Noro virus, Multi drug resistant gram negative bacteria (MDRGN), Vancomycin Resistant *Enterococcus* (VRE), *Listeria monocytogenes, Helicobacter pylori*, Sars-CoV-2

The eligible women are randomized into placebo (*GP*, 30 women), and intervention group *(GI*, 30 women). The randomization 1:1 is done in blocks of 4 mothers. The study is double blinded. The laboratory technicians who prepare the FMT product also perform the computer-generated randomization.

For promoting the study, we have set up posters and informed the staff at the maternity clinics, and used social media.

A fecal sample of 100 g and a blood sample of 15 mL are collected from the mother approximately two weeks prior to scheduled CS for screening of contagious diseases (Fig. 1). The mother divides the feces into the administered sample tubes according to written instructions. Feces for the transplant is collected into an empty tube using a spoon in the cap. The mother informs the research nurse, who delivers the tubes taken for screening to the laboratory as soon as possible, where the transplant is prepared from the fresh sample – this process is completed with a 6 h time frame.

The fresh fecal sample is screened for the presence of parasites and pathogenic viruses and bacteria (Fig. 1, Table 1). An aliquot (0.125 g) of the fresh fecal sample is used to determine the bacterial composition of the sample. Another part of this fresh sample is used as the transplant and for that purpose 1 g of feces is dissolved in 15 mL of isotonic saline and 10% glycerol, homogenized, and the 0.5 mL aliquots of the transplant are immediately frozen and stored at -80 °C [32].

At delivery, the transplant is thawed and 0.5 mL representing 3.5 mg of the mother’s fecal sample is dissolved in 5 mL of the mother’s own milk or when not available pasteurized bank milk. The sample is given orally to newborn infants born to *GI (Intervention group)* mothers within 2 h of delivery. The newborn infants born to *GP* (*Placebo group)* mothers are given 0.5 mL of isotonic saline dissolved in 5 mL of pasteurized bank milk or mother’s own milk as described earlier. The randomization is performed on the day of the scheduled CS and 0.5 mL of either transplant or placebo is delivered to the ward on ice in a black polypropylene tube for blinding purposes.

Infant exclusion criteria are Apgar score of less than 8 at 5 min of age, birth weight below 2500 g or above 4500 g, disturbances of neonatal adaptation (such as transient tachypnea of the newborn) and antibiotic treatment of the newborn before discharge. In case of a suspected infection of the newborn the randomization code can be opened.

After the first feeding the newborn is observed for potential reactions, such as increased peristalsis, diarrhea, vomiting, fever, and rash. Possible adverse events are reported to the electronic database. Before discharge, typically on postnatal day 2, a 3 mL blood sample is gathered from the newborn for the assessment of white cell count, C-Reactive Protein (CRP) and hemoglobin, and for detailed immunological analyses. The sample is taken at the routine sampling for metabolic screening.

During the hospital stay two fecal samples are collected from the newborn: on the day of birth and third day of life (Fig. 1).

From the first postnatal week on, the infant is followed in the well-baby clinic according to national protocols. As a part of the study, the parents can be in contact with the study nurse and pediatrician by telephone. Fecal samples of the infant are taken weekly for the first 4 weeks, and at 8 weeks and 3 months, by the parents. Fecal sample from the mother is taken 4 weeks postpartum. Samples are temporarily stored at − 20 °C in the home freezer before transfer in frozen form to − 80 °C. (Fig. 1).

Breastfeeding mothers give a breast milk sample at 2 and 8 weeks postpartum. Milk samples are temporarily stored at − 20 °C in the home freezer before transfer in frozen form to − 80 °C.

At 3 months postnatally, the study nurse visits the family homes. The visit is timed before the second orally given rota virus vaccination, if possible. Data from growth measurements performed at the well-baby clinic visits are gathered. Fecal samples of the infant and the mother (4 weeks post partum) stored in the home freezer are collected during the visit. A capillary blood sample of 1–1.5 mL is collected by the study nurse. The families have the possibility to meet with the study pediatrician (Fig. 1).

At 6 months of age fecal samples are collected from the mother and infant and a 3 mL blood sample is collected from the infant at a laboratory.

At 12 months of age, a follow-up visit to the study nurse takes place for all families for the assessment of growth (Fig. 1). Also, data from growth measurements performed at the well-baby clinic visits are gathered as well as vaccination status. A week prior to the visit, fecal samples of the infant and the mother are taken by the parents and stored at -20 °C in the home freezer and delivered at the visit. At the visit a 5 mL blood sample is collected. The families have the possibility to meet with a pediatrician. The families are also given a sealed envelope containing information of the allocation group.

At 24 months of age, a follow-up visit to the study nurse takes place (Fig. 1). The visit, including collecting fecal samples from mother and child, and a 5 mL blood sample from the child, is identical to the follow-up visit at the age of 12 months.

## Methods

### Fecal samples

The maternal transplant is prepared from a fresh fecal sample taken at least one week before elective CS and within 6 h of donation prepared as described [32], and frozen at -80C.

For the follow-up samples, a home sampling kit is provided for fecal samples of the mother and of the infant’s feces from diapers. The intestinal microbiota of mothers and infants is determined using 16S rRNA gene amplicon sequencing using the MiSeq sequencing [22, 23]. Fecal DNA is extracted by repeated bead beating from ca. 125 mg of fecal material [33] and processed for sequencing of the hypervariable V3-V4 region of the 16S rRNA gene using primers 341F 5′-CCTACGGGNGGCWGCAG-3′ and 785R 5’-GACTACHVGGGTATCTAATCC-3′ [15, 34] and barcoding primers from Kozich et al. [35] as explained in detail elsewhere [36]. The pooled libraries are sequenced with Illumina MiSeq platform using a MiSeq v3 reagent kit (MS-102–3003) with 5% PhiX as spike-in (Illumina).

The DNA sequences are processed and analyzed using the R-package mare [37, 38], which uses USEARCH and ASVs for read processing, and taxonomic annotation [39]. DNA extractions and MiSeq runs are processed in a time frame of 12 months and include internal reference samples and, if requested, a mock community [40, 41]. Metagenomic analysis is conducted essentially as previously demonstrated [42]. In brief, Nextera XT libraries are sequenced with Illumina Novaseq with the target of 7 Gbp of sequence per sample at the sequencing laboratory of the Institute for Molecular Medicine Finland FIMM Technology Centre, University of Helsinki. Filtered reads are assembled with Metaspades and Megahit and ORFs are predicted with Prodigal. Species level community profiling based on marker genes are done using Metaxa2. Resistance genes are characterized by mapping metagenomic reads with Bowtie2 against the CARD and Resfinder databases to search for acquired antibiotic resistance genes [42].

### Blood samples

We collect 1.5–5 mL of blood from the children depending on their age. For mass cytometry analysis, 100 μL whole blood is mixed with Whole Blood Cell Stabilizer (Cytodelics AB, Stockholm, Sweden) at a ratio of 1:1, incubated at room temperature for 10 min and transferred to a − 80 °C freezer for storage (6–9 months as per the manufacturer’s recommendation) until further processing for mass cytometry experimentation [2].

For fix/lysis of stabilized and cryopreserved whole blood sample, 100 μL sample is thawed at 37 °C followed by addition of Fix/Lyse buffer (Cytodelics AB, Stockholm, Sweden) at a blood:buffer concentration of 1:10 and incubated at room temperature for 10 min. The sample is then diluted 1:4 with Wash buffer #1 (Cytodelics AB,Stockholm, Sweden) and left to lyse for 15 min at ambient temperature. Cells are then washed twice with Wash buffer #2 (Cytodelics AB, Stockholm, Sweden), filtered through a 35 μm mesh and counted using a Bio-Rad TC20 cell counter [2].

Peripheral blood mononuclear cells (PBMC) are isolated using density gradient-based separation. 300–500 µL of blood is put in a BD Vacutainer CPT tube and centrifuged at 1600xg for 15 min followed by collection of buffy coat. Cells are frozen in freezing medium containing 50% RPMI, 30% FBS and 20% DMSO and processed as described previously [2] and transferred to a − 80 °C freezer for long-term storage awaiting analysis.

Plasma samples are obtained by centrifugation of blood samples at 2000xg for 10 min at 8–12 °C and supernatant collected and processed as described previously [2]. The samples are frozen at -80 °C post collection. Plasma protein analyses are done using Olink’s PEA assay (Olink, Uppsala, Sweden).

Humoral vaccine responses are measured at the age of 12 and 24 months as follows: concentrations of serum IgG to pneumococcal capsule polysaccharides included in the 10-valent PCV (1, 4, 5, 6B, 7F, 9 V, 14, 18C, 19F and 23F) and *Haemophilus influenzae* type b capsule polysaccharide are measured from serum samples with a fluorescent bead-based multiplex immunoassay (Luminex) as described previously [43].

Assessment of allergen-specific IgEs are analyzed with Immuno-CAP (Phadia, Uppsala, Sweden) in blood samples collected at the 12 and 24 months visits at the Laboratory of Helsinki University Hospital as described earlier [44].

### Breast milk

The samples are collected 2 and 8 weeks post-partum from breast feeding mothers. FUT2 dependent milk oligosaccharides are analyzed as described earlier [23]. In essence, skimmed breast milk samples are analyzed using MALDI-TOF (matrix assisted laser desorption/ionization—time of flight) mass spectrometry (MS) profiling and liquid chromatography to quantify 2′fucosyllactose (2′FL) and by high performance anion exchange chromatography (HPAEC) with a CarboPac PA1 analytical column coupled to a pulsed amperometry detector (ICS3000, Thermo Fischer Dionex, Sunnyvale, USA).

### Questionnaires

Data are collected on the families’ lifestyle, environmental exposures and the health of the study infants and their parents using online questionnaires, enabling monitoring and data query during data collection. Questionnaires are filled by the parents before the birth of the infant and subsequently. Parents are requested to fill in questionnaires on child’s nutrition, gastrointestinal function, and care practices weekly for the first 3 months, then monthly until 12 months of age and then at 18 months and 24 months of age. The questions change as the child grows. Illnesses, medication and use of probiotics and other dietary supplements are reported in the questionnaires. The questionnaires related to nutrition, health and background are mainly similar as in the ongoing HELMi study [45]. To enhance compliance of response rates to the questionnaire, parents receive automatic reminders via email and SMS-messages.

## Outcome

### Microbiome

The primary outcome of the study is the difference in composition of the intestinal microbiome, specifically diversity and relative abundances of Bacteroides spp., Bifidobacterium spp. as well as known pathobionts between the two groups at 3 months of age. We use beta-diversity between the microbiota profiles of the two groups calculated by PERMANOVA as described previously [46]. The analysis of the gut microbiome is performed at 12 months (including previous sequential samples) and at 24 months. Unblinding for the parents is done at 12 months and for the research group at 24 months.

### Markers of immunodevelopment

Early life immune system development follows a standardized trajectory recently described in human infants [2]. By analyzing ~ 50 markers per immune cell we are able to assess all white blood cell populations and their phenotypic and activation state. By mapping the immune development in this proposed study, we can directly compare immune developmental trajectories between children with different microbiomes.

### Markers of allergens and vaccine responses

The secondary outcomes are the differences in markers of allergy and vaccine responses at 12 and 24 months of age.

### Power analysis

The trial is designed with a planned sample of 100 mothers, allowing the final number of the subjects with an estimated 40% drop-out rate due to screening positivity to be 60 (30 in each intervention group) taking into consideration that it is likely to have drop-outs during the follow-up period.

Heterogeneity of microbiota is calculated as an index and used as the primary outcome at 3 months of age. The Standard Benjamini–Hochberg corrections for multiple testing are applied when testing multiple taxa. FDR-corrected *p*-values < 0.15 are considered statistically significant, but if needed uncorrected *p*-values are reported for clarity. 15 infants per CS group would be sufficient to detect a moderate effect size of 0.25 standard deviations for our primary outcome (power calculation for balanced one-way ANOVA test, 85% power, α = 0.05).

## Supplementary Information

**Additional file 1.**

## Data Availability

Findings from this study will be communicated with the scientific community and participants. Scientific communication will be through presentations at relevant international and domestic conferences and meetings, as well as publications in peer-reviewed journals.

## Abbreviations

CRP: C-reactive Protein
CS: Cesarean section
FMT: Fecal microbiota transplant
FUT2: Fucosyltransferase 2
GBS: Group B *streptococci*
GI: Intervention group
GP: Placebo group
VD: Vaginally-delivered
