# Supplementary material for: Transplantation of maternal intestinal flora to the newborn after elective cesarean section (SECFLOR): study protocol for a double blinded randomized controlled trial
Source: BMC Pediatr. 2022 Sep 29;22:565. doi: 10.1186/s12887-022-03609-3 (PMC9521560; doi:10.1186/s12887-022-03609-3)
Supplement: Supplementary file 1 — Additional file 1. [file 12887_2022_3609_MOESM1_ESM.docx]

| **Data category** | **Information** |
| --- | --- |
| Primary registry and trial identifying number | ClinicalTrials.gov NCT4173208 |
| Date of registration in primary registry | 21 November, 2019 |
| Source(s) of monetary or material support | Grants from sponsors mentioned below |
| Primary sponsor | Academy of Finland |
| Secondary sponsor(s) | Governmental Subsidy for Clinical Research, Suomen Lääketieteen Säätiö, Biocodex Microbiota Foundation, Foundation for Pediatric Research in Finland, Päivikki and Sakari Sohlberg Foundation, Finska Läkaresällskapet |
| Contact for public queries | Noora Carpén, MD, [noora.carpen@helsinki.fi](mailto:noora.carpen@helsinki.fi) |
| Contact for scientific queries | Otto Helve, MD, Dos THL, Team Leader, Chief Physician, otto.helve@thl.fi |
| Public title | SECFLOR-tutkimus |
| Scientific title | Transplantation of maternal intestinal flora to the newborn after elective cesarean section (SECFLOR) |
| Countries of recruitment | Finland |
| Health condition(s) or problem(s) studied | Cesarean section, development of microbiome and immune system |
| Intervention(s) | Fecal maternal transplant  Placebo comparator: isotonic saline |
| Key inclusion and exclusion criteria | Inclusion criteria: healthy pregnant woman, scheduled elective cesarean section  Exclusion criteria: Maternal exclusion criteria are gestational diabetes that requires medication, use of regular medication, travelling abroad within the last three months, antibiotic treatment within 3 months of delivery (excluding the antibiotic given immediately prior to clamping of the umbilical cord) and CS after the onset of labor (non-elective CS) |
| Study type | Interventional Allocation: randomized Intervention model: parallel assignment Masking: double blind (subject, investigator, outcomes assessor) |
| Date of first enrolment | November 2019 |
| Target sample size | 100 |
| Recruitment status | Recruiting |
| Primary outcome(s) | The difference in composition of the intestinal microbiome between the two groups at 3 months of age |
| Key secondary outcomes | The secondary outcomes are the differences in markers of allergy and vaccine responses at 12 and 24 months of age |
